# Deep learning for early detection of pathological changes in X-ray bone microstructures: case of osteoarthritis

**DOI:** 10.1038/s41598-021-81786-4

**Published:** 2021-01-27

**Authors:** Livija Jakaite, Vitaly Schetinin, Jiří Hladůvka, Sergey Minaev, Aziz Ambia, Wojtek Krzanowski

**Affiliations:** 1 School of Computer Science and Technology, University of Bedfordshire, Luton, LU1 3JU UK; 2 Pattern Recognition and Image Processing Group (PRIP), TU Wien, Wien, Austria; 3 Department of Paediatric Surgery, Stavropol State Medical University, Stavropol, Russia; 4 Fusion Radiology, Luton, UK; 5 College of Engineering, Mathematics and Physical Science, University of Exeter, Exeter, EX 4QF UK

**Keywords:** Mathematics and computing, Osteoarthritis

## Abstract

Texture features are designed to quantitatively evaluate patterns of spatial distribution of image pixels for purposes of image analysis and interpretation. Unexplained variations in the texture patterns often lead to misinterpretation and undesirable consequences in medical image analysis. In this paper we explore the ability of machine learning (ML) methods to design a radiology test of Osteoarthritis (OA) at early stage when the number of patients’ cases is small. In our experiments we use high-resolution X-ray images of knees in patients which were identified with Kellgren–Lawrence scores progressing from 1. The existing ML methods have provided a limited diagnostic accuracy, whilst the proposed Group Method of Data Handling strategy of Deep Learning has significantly extended the diagnostic test. The comparative experiments demonstrate that the proposed framework using the Zernike-based texture features has significantly improved the diagnostic accuracy on average by 11%. This allows us to conclude that the designed model for early diagnostic of OA will provide more accurate radiology tests, although new study is required when a large number of patients’ cases will be available.

## Introduction

Osteoarthritis (OA) is the most common musculoskeletal condition and a major cause of disability in older adults. It is the fifth most important cause of disability in high income countries. It is estimated that in the UK OA causes loss of 200 disability-adjusted life years per 100,000 people. New methods for early diagnostics of OA are urgently needed in order to improve patient’s treatment outcomes. Without reliable OA diagnostics new treatments cannot be developed and evaluated^1^.

Radiologists can identify the pathological changes associated with OA by analysing high-resolution knee X-ray images. Typically these changes cause the narrowing of joint space and development of bone spurs, leading to pain and impaired movement in patients. These pathologies are diagnosed in patients with developed symptoms such as joint pain^2–4^. However at early stages small pathological changes in bone microstructure can be evaluated by using a high-resolution technology such as MRI which is costly and not widely accessible. The use of advanced image analysis is expected to provide cost-efficient diagnostic solutions capable of delivering reliable estimates of pathological changes in bone microstructure^5–10^.

In many cases textures of patterns existing in images can be efficiently defined by spatial distributions of pixels^11,12^. Estimates of the pixel distribution enable patterns of interest to be represented quantitatively. In practice however the designed features can be incomplete, irrelevant, or have unexplained variations which affect the interpretation accuracy and reliability and so can cause undesirable consequences^13,14^. The desired texture features have to be capable of representing the main variations of the pixel distribution so as to explain structural changes in patterns of interest^12,15^. Specifically X-ray texture descriptors have been used for OA diagnostics^16–19^. However the texture-based features are not yet capable of delivering accurate, reliable, and reproducible diagnoses, because the results are influenced by X-ray technological conditions such as modality, exposure, blur, magnification, and projection angle. The diagnostic results are also influenced by the natural variations in the bone textures between patients of one group^20^.

Image processing techniques such as Fourier and wavelet transforms have been also used for detecting OA in X-ray images^21^. In particular the combinations of radiological features have improved the detection accuracy, although the new features are difficult to interpret as markers. The diagnostic values of the combined features were empirically tested by using Fisher-score statistics^3^.

Another approach has combined Gray-level matrix with the 2-dimensional Gabor filter to find new texture features capable of improving the detection accuracy. The diagnostic values of the newly generated features have been also empirically estimated^15^.

The designed features are expected to provide a high diagnostic value when images are rotated and have different scales. The texture features which are invariant to the rotation can be designed by using Zernike orthogonal moments. The required invariance to the image scale has been obtained by using regular geometrical moments. The high-order Zernike moments have been used for solving image classification problems^22,23^.

The use of Zernike moments for early diagnostics of OA within a Machine Learning framework has been proposed in our previous work^24^. This framework has efficiently learnt new texture features from high-resolution knee X-ray images. Although the Zernike moments were computationally efficient to provide the required invariance, additional efforts are still required in order to find new radiological markers capable of improving the detection accuracy. However the existing Machine Learning methods are still limited in delivering reliable solutions^25,26^.

The accuracy of diagnostic methods is particularly dependent on sample size of patients’ cases which have been collected and clinically verified for purposes of designing a diagnostic model. It is important to note that the sample size of patients’ cases verified at the early stage is typically small, whilst the collection of a large amount of cases is expensive and resource demanding. The most accurate results are achieved when diagnostic models are designed with representation learning which extends experts’ knowledge^27,28^. However in practice experts often cannot define an optimal framework within which a diagnostic model could be designed so as to provide a high accuracy. Representation learning allows experts to extend their knowledge by designing new model structures and features capable of increasing the diagnostic accuracy.

An early Deep Learning strategy is known as Group Method of Data Handling (GMDH) which has been proposed to learn models of “optimal” complexity required for achieving the maximal accuracy in prediction and classification^29,30^. Within the GMDH framework a multilayered model is designed from reference functions which have a small number of arguments. GMDH generates a new layer while the model’s performance increases. Such a strategy iteratively grows model connectivity and makes the GMDH strategy particularly efficient for designing models on a small data set. The GMDH-type neural networks have been efficiently used for finding solutions to the medical problems^31–33^. The GMDH framework has been successfully used in our previous work on EEG classification^34^ as well as on learning EEG features for biometric identification^35^.

In this study we aim to extend the experimental evidences that the proposed GMDH framework significantly outperforms the Machine Learning techniques in terms of the accuracy of detecting the pathological changes in the bone microstructure which are related to OA at early stages. The evidences are provided by the experiments with the texture features and the techniques such as Random Forest (RF), Support Vector Machines (SVM), and Artificial Neural Networks (ANN). The comparison is made with the techniques whose parameters were experimentally optimised. The comparative experiments were run with the Haralick features and Zernike moments. As the number of patients’ cases was small in our study, the performances were compared within the leave-one-out cross-validation. We also discuss limitations of our study and finally draw a conclusion that the proposed GMDH framework will improve the accuracy of radiology opinions in similar cases of early diagnostics of OA.

The novelty in our study is outlined as follows. The new radiological markers based on the high-order Zernike moments used within the proposed GMDH framework provide a high sensitivity to the pathological changes related to OA at the early stage when the number of patients’ cases is 40, including 20 healthy and 20 patients identified at risk of OA.

The rest of the paper is structured as follows. The proposed methodology of image representation and learning of GMDH-type neural networks is described in the section Methods. This section also outlines the secondary data used in our study for experimental validation of the proposed method. The sections Results and Discussion present the outcomes obtained on the data and finally outline the main conclusions that can be drawn from the study.

## Results

In this section we describe the main results obtained with different texture features and Machine Learning methods on the high-resolution X-ray images which are outlined in the section Data. The experiments were run with Haralick features and Zernike moments described in the section Methods.

The experiments on the X-ray data were conducted with Machine Learning techniques such as RF, SVM, ANN, and the proposed GMDH-type network. RF is a bootstrap-based method which can significantly increase the accuracy by using e.g. classification trees^36^. The performances of the above techniques were optimised, as described in the section Methods.

The main results are shown in Table 1. We can see that the Zernike moments provide a better accuracy than Haralick features for all techniques used in our study. Note that the confidence cannot be calculated within the leave-one-out cross-validation used in our study on the small data. The GMDH-type network has outperformed the RF, SVM, and ANN for both types of texture features. In comparison with the two-sample Kolmogorov-Smirnov (KS) statistic test used in the section Data, the diagnostic accuracies have been improved from 72.5 to 85% for the Lateral images and from 67.5 to 77.5% for the Medial images.

**Table 1:** Performances of the RF, SVM, ANN, and GMDH-type network using the Haralick and Zernike features.

Features	Framework	Performance, %	
		Lateral	Medial
Haralick	RF	72.5	67.5
	SVM	75.0	70.0
	ANN	72.5	70.0
	GMDH	75.0	72.5
Zernike	RF	80.0	72.5
	SVM	82.5	75.0
	ANN	80.0	75.0
	GMDH	85.0	77.5

**Figure 1:**
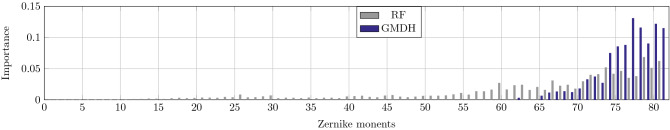
Importance of Zernike moments for the RF and proposed GMDH-type network.

In practice features used for designing a diagnostic model often make unequal contributions to the diagnostic problem. The analysis of feature importance provides new insights into the diagnostic solution. For example a new diagnostic model could be designed without a feature making a weak contribution. For models such as RF and GMDH, the importance of a feature could be defined as a frequency of its use in the designed model. Figure 1 shows the importance of the Zernike moments used in the RF (in Grey) and proposed GMDH-type network (in Black) over the 81 moments calculated for order 16, which are arranged by the increasing order. We can seen that the GMDH network has employed the moments of a higher order more frequently than the RF. Thus a conclusion can be drawn that the Zernike moments of a higher order are more informative for detection of OA at early stages.

## Discussion

Texture features are used for representation of image patterns observed as spacial distributions of pixels. Unexplained variations in the texture features lead to misinterpretation and undesirable consequences. In practice, texture features can largely vary, which makes the design of informative radiological markers problematic.

The above problems significantly affect the accuracy of early diagnostics when pathological changes in patients cannot be reliably detected within standard examinations and the amount of data which is required for designing a diagnostic solution is limited. In our previous work^24^ a solution to this problem has been suggested within a GMDH framework using texture features. The proposed solution is compared with the other Machine Learning techniques on on the patient’s cases which have been retrospectively verified at risk of OA in the high-resolution X-ray images of knees made available in the national study^37,38^. Early diagnostic cases of OA are difficult to clinically verify because the related pathological changes cannot be reliably detected by the standard radiology examination based on the Kellgren Lawrence Scoring^39^.

Our previous work has been extended in part of comparison with the Machine Learning techniques optimised during the experiments. The comparative experiments were run on the X-ray images which represent the patients’ cases including new cases of OA at the early stage. The number of the patient’s cases has been increased to 40 which is still small. The leave-one-out cross-validation used in the comparative experiments has been used for evaluating the diagnostic accuracies. The estimation of confidence intervals requires larger data sets and so was out of our research scope. Thus the main findings and insights into the early diagnostic problem are limited and cannot be directly extended to similar cases.

The first observation in our experiments was that the KS statistic test applied to the images in the Control and Case groups cannot provide a high diagnostic accuracy. Having undertaken a GMDH-based approach we have explored the texture features based on Zernike orthogonal moments which are computationally efficient and invariant to the image rotation and scaling.

The second observation was that the texture features based on the Zernike moments do not make equally important contributions to the diagnostic problem. The new radiologic features which were learnt from the small amount of patients’ data within the proposed GMDH framework have improved the diagnostic accuracy at the early stages. This is because the GMDH can iteratively increase the complexity of network connectivity at each new layer while the network performance increases. In each layer the new features are generated and those which provide best fit are selected.

In our experiments with the texture features we used different Machine Learning techniques. The experimental results show that the new radiologic markers based on the Zernike moments, which were learnt within the proposed GMDH framework, have significantly improved the diagnostic accuracy. In comparison with the baseline KS-based discrimination the diagnostic accuracy has been improved on average by 11%. Thus we conclude that the proposed method can efficiently learn new texture features from high-resolution X-ray images, which are capable of improving the diagnostic accuracy of OA at early stages.

## Methods

### Data

In our experiments we used the data collected in the national study^37,38^ which has been conducted according to the approved ethical regulations and study protocols. The data include the high resolution X-Ray images of knees in subjects. The subjects involved in the study were healthy or diagnosed at risk of OA. The samples of X-ray images as well as details of X-ray imaging are available from the study coordinators^37,38^. The referenced study particularly provides the instrumental information on X-ray imaging, such as: DX modality, Dexela Detector, $75\,\upmu {\mathrm{m}}$, $3072 \times 1944$ pixel resolution, 14 Gray-level bits.

The patients’ cases were retrospectively analysed by experts to identify the cases when the pathological changes had not yet developed and their radiology examinations based on Kellgren–Lawrence (KL) scoring^39^ were estimated at grade 1. Such cases have been further investigated in order to identify those with developed OA confirmed by the KL examination at a grade higher than or equal to 2. As the radiology study has been conducted within a given period and a defined population, the number of the patients identified to be with the progressing KL grades was small.

**Figure 2:**
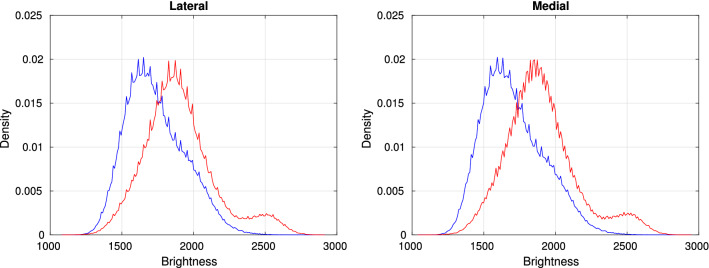
Distributions of pixel brightness values for Lateral (left) and Medial (right) images, Control (Blue) and Case (Red).

The Case (pathology) group in our study includes 20 subjects at risk of OA at the early stage. The Control group includes 20 subjects randomly selected from the healthy population. The images of both groups were taken from the Lateral and Medial compartments of the *tibia* bone. The Region of Interests (ROIs) required for our experiments have been automatically selected by using an entropy-based method^40^. Other image segmentation methods^41–43^ are capable of providing ROIs selected from high-resolution knee X-ray images. The average size of the ROI images was 150 by 150 pixels. The ROIs which were used in our experiments are available at 10.6084/m9.figshare.8303996. Figure 2 shows the distributions of the brightness values in the Lateral (left) and Medial (right) images of the Case and Control groups shown in Red and Blue, respectively. Table 2 shows the mean value and standard deviation $\sigma$ of the pixel brightness over the Control and Case groups. We can see that the mean brightness for the Case image group is higher than that of the control group in the both Lateral and Medial regions. This observation can be explained by an increasing bone density in patients of the Case group^16^.

**Table 2:** Statistics of pixel brightness in lateral and medial images.

	Lateral		Medial	
	Mean	$\sigma$	Mean	$\sigma$
Case	1735.5	205.8	1723.9	222.1
Control	1901.5	251.1	1917.2	224.0

Taking into account the above finding, we could hypothesise that the density distributions of pixel brightness in the images of Control and Case groups are significantly different. To test this hypothesis let us define the brightness distributions of the images of Control and Case groups, $C_0$ and $C_1$, respectively. Then having a distribution of an image, $C_x$, we can use the two-sample KS statistic to test the hypothesis $H_0$, that the samples $i_x$ are drawn from either the population $C_0$ or population $C_1$. The *p*-values, $p_0$ and $p_1$, are calculated for the Control and Case groups to verify the hypothesis $H_0$ at a given significance level $\alpha =0.05$. The hypothesis $H_0$ is rejected if both $p_0$ and $p_1$ are smaller than $\alpha$. In such a case the given image cannot be recognised between the groups at the given significance level.

Let the KS statistics $D_0$ and $D_1$ be the maximal distances between the empirical distribution functions estimated for an image $I_x$ and the Control and Case populations $C_0$ and $C_1$, respectively. Then we can assign the image $I_x$ to a group with the minimal distance $\min (D_0,D_1)$.

The above algorithm is used in our study as the baseline diagnostic rule. Table 3 shows the accuracy obtained with this baseline KS-discrimination on the Lateral and Medial types of images. The rejection rate shown in this table is calculated as a percentage of the images for which the hypothesis $H_0$ was rejected. At the given significance level the diagnostic results were rejected for 62.5% of the Lateral and 70.0% of the Medial images.

**Table 3:** Performance and rejection rates of KS-based discrimination for Lateral and Medial image types.

	Lateral	Medial
Performance, %	72.5	67.5
Rejection, %	62.5	70.0

### Texture features

Haralick features^11^ are based on Grey tone spatial dependencies (the Grey level co-occurrence matrix) which allow for extraction of statistical characteristics between pairs of pixels in the image. These characteristics are computed along a direction and a distance between pixel pairs. The co-occurrence matrix includes the contrast and entropy calculated for the image. The contrast estimates the difference between Grey-level values of pixels, while the entropy measures the randomness or homogeneity of the pixel distribution over the image coordinates.

Following the work^23^, Zernike moments represent a given image *f*(*x*, *y*) by a set of features which are invariant to its position, size, orientation as well as to the image rotation and scale. The desired properties are provided by moments *m* which represent the global information about the image:

1 $$\begin{aligned} m_{pq}=\sum _x{}\sum _y{x^py^qf(x,y)}, \end{aligned}$$

where $m_{pq}$ is the moment of ($p+q$)th order.

The above moments $m_{pq}$ are not orthogonal and so cannot be calculated efficiently. Zernike polynomials which are orthogonal provide efficient computation of the features. Zernike moments are calculated as the orthogonal complex polynomials $V_{nm}(x,y)$ which can be rewritten within the unit circle $x^2+y^2 \le 1$ as:

2 $$\begin{aligned} V_{nm}(x,y)=V_{nm}(\rho ,\theta )=R_{nm}(\rho )e^{(-jm\theta )}, \end{aligned}$$

where $n>=0$ are positive integers, *m* are positive or negative integers subject to the constraints: $|m| \le n$ and $n-|m|$ are even, $\rho$ is the length of a vector to a (*x*, *y*) pixel, and $\theta$ is the angle between the *x*-axis and the vector $\rho$.

The above $R_{xy}$ are the radial polynomials which are defined as:

3 $$\begin{aligned} R_{nm}=\sum _{k=0}^{(n-|m|)/2}\frac{(-1)^k(n-k)!}{k!((n+|m|)/2-k)!((n-|m|)/2-k)!}\rho ^{n-2k}. \end{aligned}$$

Zernike moments $A_{nm}$ are the projections of an image *f*(*x*, *y*) onto the orthogonal polynomials $R_{nm}$. The Zernike moment of order *n* with repetition *m* is

4 $$\begin{aligned} A_{nm}=\frac{n+1}{\pi }\sum _x{}\sum _y{f(x,y)V^*_{nm}(\rho ,\theta )}. \end{aligned}$$

Additional details of the Haralick features and Zernike moments used in our study are provided in the section Experimental Settings.

### GMDH-type neural networks

The GMDH-type Deep Learning adopted in our study is based on heuristic-based optimisation of multilayer neural networks with a polynomial activation function. GMDH generates new features and grows up a network connectivity on given training data^29^. The heuristic optimisation is based on so-called “external” criteria aimed to select networks which are fitted best to the training data, This allows GMDH to efficiently prevent networks from overfitting, which affects the network ability to generalise and predict unseen data. New features are generated in each new layer by combining the input variables and the outputs of neurons from the previous layers. The number of layers grows while the network performance increases. When the network connectivity becomes “optimal”, the generalisation ability reaches the maximum value which can be achieved within the defined activation function and selection criteria.

In GMDH-type networks activation functions are defined as short-term polynomials which can be linear or non-linear, $y=g({x};{w})$, where *x* is the input vector, and *w* is the coefficient vector. Using a given activation function *g*(*x*; *w*), the GMDH generates a new feature vector ${x}=(x_{i_1},x_{i_2})$ for *K* neurons at the layer $r=1$, with indexes $i_1 \ne i_2,i_1=1,\dots ,m,$ and where $K=m(m-1)/2$ is the number of pairwise combinations for the given *m* input variables. The outputs of neurons at the *r*th layer, ${y}_i$, are written as

5 $$\begin{aligned} {y}_i^{(r)}=g(x_{i_1},x_{i_2};{{\hat{w}}}^{(r)}_i),\quad i=1,\dots ,K. \end{aligned}$$

Given a set of the indexes *A*, the coefficient vectors ${{\hat{w}}}^{(r)}$ are fitted to the data $[{X}({A})\ | \ {\mathring{y}}({A})]$ for each neuron. The results are numerically stable as the number of data samples included in the set *A* is larger than the number of variables *m* and the columns $i_1,i_2$ of the matrix ${X}({A},[i_1,i_2])$ are not correlated. Thus the fitted weights ${{\hat{w}}}$ are:

6 $$\begin{aligned} {{\hat{w}}}^{(r)}= [{1 \ X({A}},[i_1,i_2])]^{-1} {\mathring{y}}({A}), \end{aligned}$$

where 1 denotes the unit vector.

Having the outputs ${y}_i$ on the entire data *X*, the regularisation errors $\Delta$ can be calculated as follows:

7 $$\begin{aligned} \Delta _i=\Vert {y}_i-{\mathring{y}} \Vert . \end{aligned}$$

The errors are sorted in ascending order, and then the first *F* neurons with the lowest errors are selected for the next layer. The neurons with correlated outputs are excluded from the selection ${y}_{i_1},\dots ,{y}_{i_F}$. and so the number of selected neurons at the *r*th layer can be $F_r$: $1 \le F_r \le F$. The neurons at the next layers $r+1$ are generated by applying the activation function *g* to extended data ${Z}^{(r)}=[{Y}^{(r)}\ | \ {X}]$, which include the outputs of the selected neurons ${Y}^{(r)}=[{y}_{i_1},\dots ,{y}_{i_{F_r}}]$ and the input data *X*. Thus the matrix ${Z}^{(r)}$ contains $m_r=F_r+m$ columns.

Similarly, the coefficient vectors ${{\hat{w}}}^{(r+1)}$ are estimated for the $i_1,i_2$ columns of the matrix ${Z}^{(r)}$. The outputs of neurons ${y}^{(r+1)}$ are then calculated as follows:

8 $$\begin{aligned} {y}_{i}^{(r+1)}=g({z}^{(r)}_{i_1},{z}^{(r)}_{i_2};{{\hat{w}}}^{(r+1)}_i),i=1,\dots ,K_r, \end{aligned}$$

where $i_1 \ne i_2;i_1=1,\dots ,m_r,i_2=1,\dots ,m_r$, and $K_r=m_r(m_r-1)/2$ are the number of pairwise combinations for the $m_r$ columns.

GMDH generates the new layers and the network grows while the number $F_r$ exceeds a given threshold. The pseudocode of the described GMDH algorithm is outlined below.

### Learning of GMDH-type neural networks

The main steps of learning a GMDH-type network are represented by Algorithm 1. The Algorithm defines the training data $[{X}\ | \ {\mathring{y}}]$ and the number of neurons *F* (or ”freedom” of choice) to be selected for new layers. The first layer $r=1$ is generated and then neurons which are best fitted to the data are selected for the next layer. The algorithm stops if the number of the selected neurons, $F_r$, becomes less than the given threshold $F_0$.

The procedure InitiateNet assigns the network parameters *Rn*, *Ln*, *In*, *Wn*, and *Zn*. Here *Rn* are the neuron indexes *r*, *Ln* are the neurons errors, *In* are the inputs $i_1,i_2$ of neurons, *Wn* are the coefficient vectors of neurons, and *Zn* are the outputs of the selected neurons.

In GenerateFirstLayer the algorithm generates the first layer of neurons with the inputs $x_{i_1},x_{i_2}$. The neurons are adjusted to the data and the network parameters are updated in part of $\Delta$, ${{\hat{w}}}$, and *y*.

The procedure GenerateNewLayer defines the indexes of neurons at the previous layer. Then the number $F_r$ and the indexes $A_1$ of the selected neurons are defined. For the given inputs $z_{i_1},z_{i_2}$ the new neurons are generated according to the procedure GenerateFirstLayer.

In SelectBest the algorithm generates the neurons at the given layer *r*. The generated neurons are sorted out by the regularisation errors stored in *Ln* in the ascending order. The first $F_r$ neurons with the lowest errors are selected for the new layers. The selection does not include the neurons with the correlated outputs. The procedure returns *false* if the number of the selected neurons is below the threshold $F_0$. Otherwise the procedure returns *true* to further grow the GMDH-type network.

The procedure UpdateNet fits the coefficient vector ${{\hat{w}}}$ to the given input *U*. The regularisation error $\Delta$ is evaluated for the fitted neurons and then the network parameters *Net* are saved for the next layers.

The GMDH algorithm generates the new layers whilst the network performance is increased by a defined value. Otherwise the algorithm stops and the grown network is represented by the matrix *Net*. 
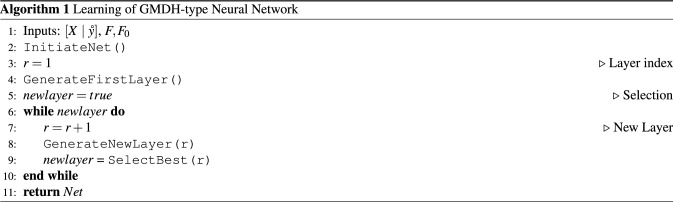


### Experimental settings

Performances of RF, ANN, SVM as well as GMDH-type networks are dependent on their parameters which have to be optimised in a “try-and-see” way during experiments on given data^44^. The diagnostic performance was estimated using the leave-one-out cross-validation method which is typically used when sample size is small^45^.

It is important to note that standard Principal Component Analysis cannot be efficiently used for data dimensionality reduction when the data sets are small. This is because the principal components calculated on a small data set are subject to large variability^46^.

In our comparative experiments the maximal performances of the RF, ANN, and SVM have been achieved with the following settings.

1. The best RF was with the number of classification trees $=200$, the minimal number of samples at terminals $=3$, attribute rate $=0.8$, and sample rate $=0.7$
2. The best ANN was with the number of hidden neurons $=\{7,12\}$, the training metod=Levenberg-Marquardt, the learning rate $=\{0.2,0.6\}$, early stopping rule, activation $=$ logsig
3. The best SVM was with a Radial Basis Function kernel and a gamma optimised on the cross-validation.

The maximal performance of the GMDH has been experimentally found with the following parameters.

1. The freedom of choice $F=m$, where *m* is the maximal number of texture features
2. The minimal number of neurons $F_0=2$
3. The activation function $y=\alpha _0+\alpha _1x_1+\alpha _2x_2+\alpha _3x_1x_2$
4. The size of training data was 31, and 8 data samples were used for the external estimation in each of the 40 rounds of the leave-one-out cross-validation.

The Haralick features were used with the three textural parameters extracted from the grey level co-occurrence matrices^11,47^. Table 4 shows the Haralick texture features used in our experiments.

**Table 4:** Haralick texture features.

Nos.	Name
1	Angular second moment (energy)
2	Contrast
3	Correlation
4	Variance
5	Inverse difference moment (homogeneity)
6	Sum Average
7	Sum Variance
8	Sum Entropy
9	Entropy
10	Difference Variance
11	Difference entropy difference entropy
12	Information measure of correlation I
13	Information measure of correlation II
14	Maximal correlation coefficient

The Zernike moments were calculated for order 16 so as to generate 81 features. In our experiments the real part of the moments were taken as the texture features.

## Regulation statements

The authors make the following statements: The knee X-Ray images selected for our experiments have been collected in the national study^37,38^ conducted according to the approved ethical regulations and study protocols. The study was reviewed and approved by the National Committee for Data Protection (Comissão Nacional de Proteção de Dados) and by the NOVA Medical School Ethics Committee. Ethical Committees of Regional Health Authorities (ARS) also reviewed and approved the study.

All methods used in our study were performed in accordance with the relevant guidelines and regulations. The experiments described in our paper have been conducted on knee X-Ray images of participants provided informed consent within the above mentioned national study.
